# Review of Anti-Inflammatory Herbal Medicines

**DOI:** 10.1155/2016/9130979

**Published:** 2016-05-10

**Authors:** Mona Ghasemian, Sina Owlia, Mohammad Bagher Owlia

**Affiliations:** ^1^School of Pharmacy, Shahid Sadoughi University of Medical Sciences, Yazd, Iran; ^2^School of Medicine, Shahid Sadoughi University of Medical Sciences, Yazd, Iran

## Abstract

Medicinal plants and their secondary metabolites are progressively used in the treatment of diseases as a complementary medicine. Inflammation is a pathologic condition that includes a wide range of diseases such as rheumatic and immune-mediated conditions, diabetes, cardiovascular accident, and etcetera. We introduce some herbs which their anti-inflammatory effects have been evaluated in clinical and experimental studies.* Curcuma longa, Zingiber officinale, Rosmarinus officinalis, Borago officinalis*, evening primrose, and Devil's claw are some of the introduced medicinal herbs in this review. Since the treatment of inflammation is not a one-dimensional remedy, this review tries to reach a multidimensional therapeutic approach to inflammation with the help of herbal medicine and modification in lifestyle.

## 1. Introduction

Inflammation is a defense response of our body to hazardous stimuli such as allergens and/or injury to the tissues; on the other hand, uncontrolled inflammatory response is the main cause of a vast continuum of disorders including allergies, cardiovascular dysfunctions, metabolic syndrome, cancer, and autoimmune diseases imposing a huge economic burden on individuals and consequently on the society [1]. There are various medicines for controlling and suppressing inflammatory crisis; steroids, nonsteroid anti-inflammatory drugs, and immunosuppressant are the practical examples of these medications which are associated with adverse effects while in practice our goal is to apply minimum effective dose by the highest efficacy with the least adverse effects. Thus, we need to apply natural anti-inflammatory factors within medication therapy to achieve increased pharmacological response and the lowest degree of unwanted side effects [1, 2]. Herbal medicines are promoting subjects in medicine and, of course, we have to increase our knowledge about them. Complementary, alternative, and traditional medicines are the pivotal source of herbal medication guidance, but surely modern medicine must prove these guidelines through scientific methods before using them in practice. In this review, we have endeavored to assess the plants and the most clinical evidence of their anti-inflammatory effects.

## 2. Methods

In this study, all the data were gathered from search engines as follows: PubMed, ScienceDirect, and Google Scholar.

We have used these keywords “anti-inflammatory”, “plant”, “herb”, and “herbal medicine” for searching in these databases.

All the references which were used to publish this review article were written in English and from the standpoint of the time interval, they belonged to 1980 to the present. The entire articles relating to our goal were collected and classified based on the level of evidence, where systematic reviews and randomized control trials (RCT) have possessed the highest values. Open-label, cohort, case-control, case series, preclinical, in vivo, ex vivo, and in vitro studies have less importance than the first two, respectively.

It is obvious that each subject that we have found which has higher valuable studies, such as RCT in association with that, has received high priority for mentioning in this literature.

### 2.1. *Curcuma longa*

*Curcuma longa* (common name is Turmeric in English, زردچوبه in Persian, cúrcuma in Spanish, 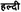 in Hindi, and عقدة  الصفراء in Arabic) is an Indian indigenous plant [3]. The most important secondary metabolite of* C. longa* is curcumin, which is responsible for anti-inflammatory effect of this plant [4].

Many clinical trials have been done for proving the anti-inflammatory effect of curcumin. Their results suggest that curcumin can be effective in improving inflammation of rheumatoid arthritis (RA) and reducing clinical manifestation of RA, such as joint swelling and morning stiffness in comparison with phenylbutazone which is used as a positive control [5]. Also, curcumin was tested in patients with anterior uveitis; after 2 weeks, exhaustive remission occurred [6]. The effectiveness of curcumin in patients with dyspepsia and/or gastric ulcer was proved by another clinical trial. In this study, subjects experienced remission after 12 weeks (maximum) [7]. Curcumin is beneficial in irritable bowel syndrome (IBS) treatment [8] and also works as a reducing agent to delayed graft rejection (DGR) after kidney transplant surgery [9]. Curcumin likewise has a beneficial effect in inhibition of inflammatory bowel disease (IBD) and reduction in sedimentation rate in patients who suffered from IBD [10]. It is also proven to be beneficial in maintaining amelioration of ulcerative colitis [11] and psoriasis (by the selective prohibition of phosphorylase kinase) [12].

### 2.2. *Zingiber officinale*

*Zingiber officinale* (common name is ginger in English, زنجبیل in Persian, 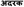 in Hindi, and الزنجبيل in Arabic) is a native plant from south-east Asia [13].

Oral administration of* Z. officinale* extract has shown different and inconsistent effects, depending on the quantity of consumption. Although administration of squeezed ginger extract to mice one time or twice has elevated the tumor necrosis factor-*α* (TNF-*α*) in peritoneal cells, long-term consumption of the extract has increased the serum corticosterone level and has reduced proinflammatory markers [14].* Z. officinale* was also tested in type 2 diabetic patients with low-grade inflammation; after 2 months of treatment, serum level of TNF-*α* and high-sensitivity C-reactive protein (hs-CRP) were decreased definitely [13]. In patients with osteoarthritis, ginger had not only efficacy in pain improvement identical to Diclofenac 100 mg but also no side effects [15]. Ginger extract has been compared to Ibuprofen and Indomethacin in OA patients; the results have exerted improving function of Ibuprofen, Indomethacin, and ginger extract equally in pain score [16–18]. Ginger powder has had ameliorative effect in musculoskeletal and rheumatism patients through inhibiting cyclooxygenase and lipoxygenase pathway in synovial fluid [19].

### 2.3. *Rosmarinus officinalis*

*Rosmarinus officinalis* (common name is Rosemary in English, رزماری in Persian, Romero in Spanish, 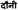 in Hindi, and روزماري in Arabic) is native in the Mediterranean area [20].

In an open-label trial, the effects of rosemary extract have been assessed in patients with osteoarthritis (OA), rheumatoid arthritis (RA), and fibromyalgia during 4 weeks; hs-CRP (an index for inflammation presence) was decreased noticeably in patients who had demonstrated augmentation in this index; by the way, reduction in inflammation related to pain score was observed during the treatment but remission has not occurred in fibromyalgia scores [21]. There is evidence that confirms anti-inflammatory potential of* R. officinalis* in molecular scope; according to this, rosmarinic acid could disturb complement system activation easily by inhibiting C3b attachment; the dose required for making this effect is very low (34 *µ*M) [22]. Furthermore, rosemary's extract has shown gastroprotective action against gastric ulcer, even better than Omeprazole; this advantage is because of inhibition activity of rosemary in neutrophils infiltration and reduction in proinflammatory mediators: TNF-*α* and IL-1 [23]. Nevertheless, in another preclinical study on rats, high dose of rosemary extract (500 mg/kg) has reduced testosterone and spermatogenesis that led to infertility [24]. This plant has had topical anti-inflammatory in wound healing in mice [25]. Carnosic acid in* R. officinalis *has interacted with CYP3A4 and CYP2B6 substrate and likewise has had toxicity in human hepatocyte with EC_50_ value identical to Tamoxifen [26].

### 2.4. *Borago officinalis*

*Borago officinalis* (common name is Borage in English, گل  گاوزبان in Persian, Borraja in Spanish, and لسان  الثور-حمحم in Arabic) is a member of Boraginaceae family and is native in European area and north of Africa [27].

This plant is a rich source of gamma linoleic acid (GLA), which contains 25% of GLA, by elevating prostaglandin-E (PGE) level that leads to cyclic adenosine monophosphate (cAMP) augmentation; GLA could count as a strong suppressor of TNF-*α*. The mechanism mentioned above can clarify the anti-inflammatory effect of borage oil in rheumatoid arthritis (RA) [28]. Regarding this pathway, borage has contraindication during pregnancy because of the miscarriage risk [28]. Antirheumatoid arthritis's potential of borage seed oil was assessed in 2 RCT as follows: in the first study, 1.4 g/day borage seed oil has been compared with placebo in RA patients; 36.8% amelioration occurred in the treatment group at the end of 6-month therapy. In the second study, 2.8 g/day of borage seed oil was taken by patients during 6 months; at the end of treatment, the amelioration percent of RA manifestation was noticeable: 64% in the treatment group compared with 21% in the control group [29]. Likewise, the anti-inflammatory effect of borage oil was tested in patients with atopic dermatitis. 12 clinical trials were performed to evaluate the effectiveness of this herb in ameliorating in atopic dermatitis. 5 of those have proved the anti-inflammatory effect and 2 of those have recorded improving in some patients, although in the rest 5 trials there has not been any observation for remission [30].

### 2.5. *Oenothera biennis* (Evening Primrose)

*Oenothera biennis* (common name is evening primrose in English, گل  مغربی  in Persian, Onagri in Spanish, 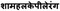 in Hindi, and زهرة  الربيع  المسائية in Arabic) is a member of Onagraceae family which is native in Central America [31].

GLA, linear aliphatic alcohols (e.g., Tetracosanol), and phenolic compound (ferulic acid) are the active components of evening primrose oil which have had protective roles against proinflammatory markers [32]. This oil has sterols such as *β*-Sitosterol and Campesterol that have had modulator effect on nitric oxide (NO), TNF-*α*, IL-1*β*, and thromboxane B2 (TXB2) leading to suppressing COX-2 gene expression; because of these reasons, the primrose oil has a greater anti-inflammatory effect than borage oil [33]. The effectiveness of evening primrose oil with hemp seed oil has been clinically assessed in multiple sclerosis (MS) patients. Patients with MS (a chronic inflammatory disorder) have randomly taken hemp seed/evening primrose oil and placebo. Significant reduction in IFN-*γ* and IL-17 has occurred in the treatment group. The relapse rate of the disease has been also alleviated in the treatment group; this study has shown the immunomodulatory impression of these oils and their components [34]. In an RCT on RA, researchers have recorded subjective improvement and reduction in using NSAIDs without any improvement in clinical measurements [35]. Likewise, patients have demonstrated remission in morning stiffness with no clinical changes in articular index or pain [36]. And no significant amelioration in target therapy group was the main outcome of a clinical trial on 18 patients with RA after 12 weeks [37].

### 2.6. *Harpagophytum procumbens* (Devil's Claw)

*H. procumbens* (common name is Devil's claw in English, پنجه  شیطان in Persian, Garra del Diablo in Spanish, and مخلب  الشيطان  in Arabic) is a member of Pedaliaceae family [38]. Among its abundant metabolites, Harpagoside has been substantiated as an anti-inflammatory component [39]. Root's extract of Devil's claw has been claimed to possess inhibition potential of NO, inflammatory cytokines (IL-6, IL-1*β*, and TNF-*α*), and PGE_2_, as well as prevention of arachidonic acid metabolism and eicosanoid biosynthesis, leading to COX-2 inhibition and reducing inflammation [40–42]. In another preclinical study, devil's claw has shown no efficacy in improving carrageenan-induced edema in the hind foot of the rat [43]. Over an RCT, the effectiveness of Devil's claw in osteoarthritis remission has been assessed. At the end of treatment period, anti-inflammatory effects of* H. procumbens* have been observed [44]. In contrast, in a pilot study which has been carried out on patients who have suffered from arthritic disease (RA and psoriatic arthropathy), researchers have not observed any remission or subjective and objective improvement with 410 mg TDS of* H. procumbens'*s liquid extract after 12 weeks [45]. Gastrointestinal upset is the main side effect of this plant which leads to contraindication in patients with gastric or duodenal ulcers, gallstone, and diabetes [38].

### 2.7. *Boswellia serrata*

*Boswellia serrata* (common name is Indian Olibanum in English, کندر  in Persian, 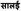 in Hindi, and الـلُّبَّـان  in Arabic) is an oleo gum resin of* Boswellia* tree, which is native in India [46].

Efficacy of* Boswellia serrata* extract in patients with osteoarthritis has been substantiated; dramatic alleviation in the frequency of joint swelling and pain and augmentation in joint flexibility and walking distance have been observed at the end of treatment period [46]. Likewise, a significant reduction in erythrocyte sedimentation rate (ESR), morning stiffness, and NSAID administration requirement during therapy has occurred in rheumatoid arthritis patients within another clinical trial [47]. In one pilot study which has been carried out on patients with chronic polyarthritis, no significant remission has been observed in patient's manifestations after 12 weeks of therapy with extract of* B. serrata*; just minor attenuation in NSAIDs requirement has been recorded [29]. Collagenous colitis is an inflammatory bowel disease (IBD) and* B. serrata* has been clinically effective in the process of ameliorating this disease in target therapy group compared to the placebo group [48]. The combination of* B. serrata* with* C. longa* and* Glycyrrhiza glabra* has been effective on improvement of asthmatic patient's symptoms; also, in this study, treatment group has demonstrated significant diminishing in plasma level of leukotriene C_4_ (LTC_4_), NO, and malondialdehyde after 4 weeks [49]. Modulating in inflammatory mediators (TNF-*α*, IL-1*β*, IL-6, IFN-*γ*, and PGE2) by* B. serrata* extract has been proved in in vivo and in vitro studies [50, 51]. Boswellic acid is the main component of this gum which can inhibit C3 convertase and suppressed classic pathway of complement system [52, 53]. Likewise, it has had topical anti-inflammatory impress as well as systemic effects [54].

### 2.8. *Rosa canina*

*Rosa canina* (common name is Dog rose in English, نسترن  وحشی in Persian, escaramujo-tapaculo in Spanish, 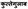 in Hindi, and ورد  الکلب-ورد  السياج- النسرين in Arabic) is a member of Rosaceae family [55].

The effectiveness of rosehip has been assessed in OA and RA patients. The outcomes of these studies were as follows: the patients, who have suffered from OA, have experienced alleviation in pain, rescue medication consumption, and stiffness and a significant reduction in CRP which have been observed after treatment with this plant [56, 57]. It should be noted that anti-inflammatory effect of rosehip refers to the seed, but not its shell. The latter claim has been substantiated via two clinical studies which have been done on OA patients [56, 58]. Likewise, rosehip powder has reduced ESR and improved quality of life in RA patients; thus, it might be used as a supplement besides the standard treatment of RA [59]. In contrast, 10 g of rosehip powder per day, during 1 month, has no anti-inflammatory effect on patients with RA [60]. The ethanol extract of rosehip was fractioned by some solvents with different polarity; ethyl acetate and butanol fraction have had anti-inflammatory effects in delayed phase of inflammation process through inhibition of PGE1 in mice [61]. Since n-hexane and dichloromethane extracts of this plant's fruit have had a downregulatory effect on COX-1, COX-2, and LTB_4_, these fractions are rich sources of unsaturated fatty acids [55]. Galactolipid is an active component in rosehip powder which its NO inhibitory potential has been confirmed through laboratory and in vitro studies [62, 63].

### 2.9. *Urtica dioica*

*Urtica dioica* (common name is stinging nettle in English, گزنه  in Persian, Ortigamayor in spanish, and القراص  الكبير  in Arabic) is a member of Urticaceae family [64].

Nettle leaf has been investigated to prove its anti-inflammatory effect in a pilot study. 50 mg Diclofenac per day was administered to patients with acute arthritis together with 50 mg infusion of* Urtica dioica *orally. This remedy has caused remarkable attenuation in CRP level and some patients' complaints for 200 mg Diclofenac per day; according to these outcomes,* U. dioica* when combined with NSAIDs have an outstanding synergistic effect [65]. Topical effectiveness of nettle leaf has been assessed in osteoarthritis of thumb through RCT; significant alleviation in pain, stiffness, and anti-inflammatory and analgesic therapy requirements have been observed [66]. The combination of nettle leaf with rosehip and willow bark has suppressed IL-1*β* and COX-2 in chondrocytes. In this in vitro study, chondroprotective and anti-inflammatory effects of this botanical extract have been proved [67]. Leaf extract of* U. dioica* has had inhibitory potential on proinflammatory transcription factor NF-*κ*B (scientific studies have shown elevation in NF-*κ*B in synovial fluid of RA patients) [68]. This extract has had anti-inflammatory potential in allergic rhinitis by the following pathways: antagonizing H_1_-receptor, reducing of PGD_2_ production (allergy specific prostaglandin), and inhibitory effect on mast cell tryptase [69].

### 2.10. *Uncaria tomentosa*

*U. tomentosa* commonly known as cat's claw in English, uña de gato in Spanish, پنجه  گربه in Persian, and مخلب  القط  in Arabic. It belongs to Rubiaceae family and it is an indigenous plant in Amazon and Central America forests [70, 71].

The efficacy and safety of this plant in improving OA of the knee have been tested on 45 patients who have been divided into 2 groups (placebo and active); the active group has demonstrated some degrees of remission after 4 weeks by inhibiting TNF-*α* and diminishing PGE_2_ production [72]. In a 24-week double-blind placebo-controlled trial which has been performed for evaluating the effect of high purified extract of* U. tomentosa* in RA patients, this extract has been administered along with Sulfasalazine or Hydroxy chloroquine; modest benefit of this herb in alleviating pain, swelling, and tenderness of joint has been shown in the treatment group in comparison with the placebo group [73]. There is a report of* U. tomentosa* causing remarkable remission in enteritis in rats which has been observed [74]. Edible extract of cat's claw has had protective action against respiratory inflammation in mice [75]. Pivotal mechanism of cat's claw is inhibition of iNOS and NF-*κ*B expression that in turn have downregulated TNF-*α*, IL-1*α*, 1*β*, 10 and 17 successively. Also, little inactivation effect on COX-1 and COX-2 has been expressed through an in vivo study [70, 71, 74]. This plant's bark has demonstrated anti-inflammatory action exactly the same as dexamethasone in an animal model, while it has attenuated about 40% of IL-4 while dexamethasone has not [76].

### 2.11. *Salvia officinalis*

*Salvia officinalis* (commonly known as sage in English, مريم  گلی in Persian, salvia in Spanish, and قصعين  طبي in Arabic) is a member of Lamiaceae family [77].

Carnosol and carnosic acid are phenolic diterpenes which have had anti-inflammatory activity [78]. These two components could have inhibited PGE_2_ production via microsomal PGE_2_ synthase-1 inhibition [79]. Chloroform extract of sage leaves has shown atopic anti-inflammatory effect in mice [80]. However, sage essential oil has not shown any immunomodulatory effect in mice which had underwent cyclophosphamide-mediated immunosuppression [81]. It is also worth mentioning that Halicioglu et al. have reported generalized tonic-clonic seizures following accidental exposure to sage oil in a newborn and a child [82].

### 2.12. *Ribes nigrum*

*Ribes nigrum* (common name is blackcurrant in English, آنگور  فرنگى  سياه in Persian, Casis in Spanish, and الكشمش  الأسود in Arabic) oil is a rich source of n-6 polyunsaturated fatty acid (PUFA), *γ*-linoleic acid, and *α*-linoleic acid [83].

In one clinical trial which has been fulfilled on RA patients during 6 weeks, researchers have investigated the effect of blackcurrant oil (BCO) on patients; outcomes were as follows: attenuation in morning stiffness in the experimental group and reduction in proinflammatory mediators including IL-1*β* and TNF-*α* in peripheral blood monocytes [84]. After 24 weeks of treatment period with BC seed oil, disease activity symptoms of RA patients have been reduced. Overall, no significant differences in clinical signs and symptoms have been recorded between the placebo and the case group [85]. Also, BC seed oil has a moderate reinforcement effect on the immune response and inhibitory effect on the PGE_2_ biosynthesis in 40 healthy volunteers older than 65 years [83]. In another clinical study, 12 healthy subjects have consumed BC oil; attenuating in LTB_4_ biosynthesis via polymorphonuclear-neutrophil (PMN) and increasing of dihomo-*γ*-linoleic acid in PMN's phospholipids have been observed [86]. BC skin extract could reduce heat shock protein (HSP70 and HSP90), COX-2, and NF-*κ*B expression in rats which were under diethylnitrosamine (hepatocarcinogen) exposure [87].

### 2.13. *Persea americana*/*Glycine max*

*Persea americana* (common name is Avocado in English, آووکادو  in Persian, árbol in Spanish, 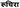  in Hindi, and  الزِبدِيّة- الأفوكاتة in Arabic) is a native fruit in Central America [88] and belongs to Lauraceae family.* Glycine max* (common name is soybean in English, سویا  in Persian, soja in Spanish, 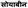 in Hindi, and فول  الصويا in Arabic) is a member of Fabaceae family, native to East Asia.

In a prospective multicenter, 3-month randomized control trial, 153 OA patients have been enrolled and treated with Avocado/soybean unsaponifiables (ASU) along with NSAID; after 45 days of therapy, NSAID requirement has diminished but no significant changes have occurred in patients' pain scores [89]. In three clinical trials which have been carried out on OA patients, the effectiveness of ASU has been assessed. Two of them have demonstrated reduction in Lequesne's functional index (LFI), pain, and disability; likewise, more than 50% attenuation in NSAID requirement has been observed in 71% of patients in the case group versus 36% in the control group, but in the last trial, no intergroup changes have been reported in joint space width (JSW) which has been considered as primary endpoint and no amelioration has been reported in clinical investigations [90]. During 3 years of follow-up of the hip in OA patients taking ASU, no improvement in JSW has been recorded, but 20% prevention of JSW exacerbation has occurred [91]. ASU also has been administrated to 100 patients with linear scleroderma and morphea; this study has shown a beneficial effect of ASU in preventing atrophy, deformity, and contracture, if the treatment with ASU has been initiated at an early stage of the disease [92]. Topical and dietary administrations of Avocado and soybean extract have been assessed in patients with mild to moderate vulvar lichen sclerosus (VLS). At the end of 24 weeks of treatment period, main sign and symptom of disease have been diminished significantly [93].

### 2.14. *Elaeagnus angustifolia*

*E. angustifolia* (common name is Oleaster in English, سنجد in Persian, and  الخلاف  ضيق  الأوراق-الزيزفون  السوري  in Arabic) is a member of Elaeagnaceae family [94].

The effectiveness of Oleaster in the treatment of oral lichen planus (OLP) lesion has been evaluated in an RCT with 28 patients. Seventy five percent and 50–75% attenuation in pain and lesion size, respectively, have been observed in the case group [94]. In another randomized clinical trial which has been carried out on 90 knee OA female patients, a significant attenuation in TNF-*α* and matrix metalloprotein-1 (MMP-1) (proinflammatory mediators) and alleviation in IL-10 (an anti-inflammatory cytokine) have been reported in active therapy group [95]. Oleaster extract has demonstrated an anti-inflammatory effect in an animal model but this effect was not significant in comparison with sodium salicylate [96]. Aqueous extract of this fruit has shown anti-inflammatory properties in mice through COX-1 and COX-2 inhibition; the evidence has exerted no correlation between corticosterone level and that of anti-inflammatory action [97].

### 2.15. *Vaccinium myrtillus*

*Vaccinium myrtillus* (commonly known as bilberry in English, بلوبرى  آروپایى in Persian, arándano in spanish, العنبية  الآسية in Arabic) is a member of vaccinium family [98].

In a randomized clinical trial, which has been carried out on 27 patients with metabolic syndrome who have received 400 g fresh bilberry daily, outcomes have been reported as follows: diminishing in hs-CRP, IL-6, and IL-12 and circulating LPS concentration in the active group [99]. Bilberry has caused remission in 63.4% of 13 ulcerative colitis patients after 6 weeks and significant reduction in mayo score and fecal protection level has occurred [100]. No changes have been observed in anti-inflammatory peptides (monocytes chemotactic protein-1) of diabetic patients after one capsule of concentrated bilberry extract (36% w/w anthocyanins) administration per day [101].

### 2.16. *Olea europaea*

*Olea europaea* (commonly known as Olive in English, زیتون in Persian, Olivera in Spanish, 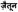 in Hindi, and الزيتون in Arabic) is a species of Oleaceae family [102].

The positive effect of extra virgin olive oil (EVOO) on modulating postprandial plasma lipopolysaccharide, proinflammatory cytokines, TXB_2_ and LTB_4_, and diminished performance in risk of coronary heart disease has been demonstrated in healthy individuals and metabolic syndrome patients [102, 103]. Oral olive oil has accelerated wound healing process and has alleviated hospitalizing duration in deep second-degree and more burn wound patients in comparison with sunflower oil (SFO) [104]. Also, disease activity index and tumor incidence of ulcerative colitis-associated colorectal cancer and proinflammatory cytokines in mice have been alleviated after EVOO enriched diet consumption compared with that of SFO-fed mice [105].

## 3. Conclusion

The amount of the plants which have been asserted to possess anti-inflammatory effect is so much that evaluating all of them is out of the scope of this paper; thus, we have sufficed to mention the herbs about which there is more evidence.

**Figure 1:**
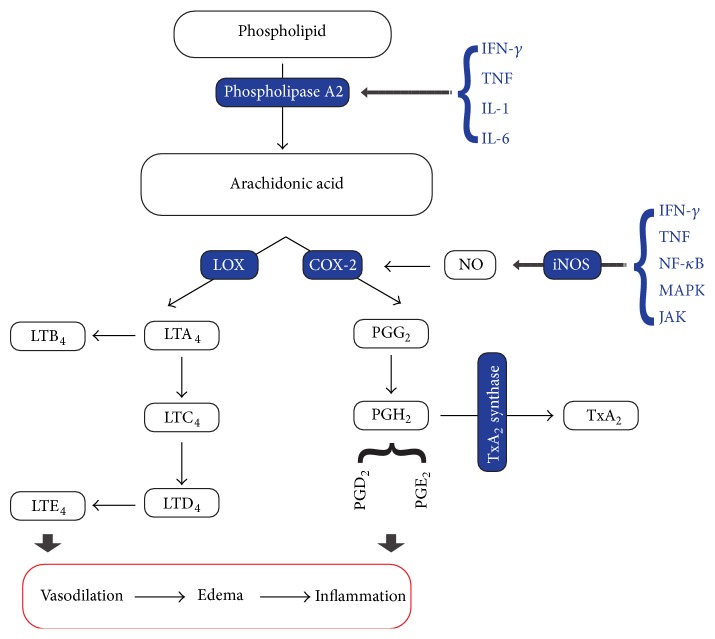
Inflammation pathway. COX, cyclooxygenase; LOX, lipoxygenase; PG, prostaglandin; LT, leukotriene; TX, thromboxane; NO, nitric oxide; iNOS, inducible NO synthase; IFN, interferon; TNF, tumor necrosis factor; NF-*κ*B, nuclear factor-*κ*B; MAPK, mitogen activated protein kinase; JAK, janus kinase; IL, interleukin.

Herbal medicine is one of the most important aspects of complementary medicines. There are many studies which have been asserted the role of several herbs in inflammation remission. We introduce some herbs which their anti-inflammatory effects have been evaluated in clinical and experimental studies; of course, clinical data is more reliable than others; among our research data, the* Curcuma longa* had the most clinical evidence about different inflammatory disorders such as RA, uveitis, and IBD. Also, other listed herbs have demonstrated good performance in clinical and experimental anti-inflammatory tests. Inflammation process has various mechanisms and numerous treatment methods consequently. Plenty of cytokines participate in enzyme activation (such as phospholipase A_2_), mediator release, fluid extravasation and vasodilation, cell migration, and finally tissue damage which generally have been named inflammation (Figure 1). Biochemical outcomes of the experimental studies clearly show the potential role of herbs in activation or inhibition of proinflammatory cytokines (Table 1), although more clinical studies with larger participants and meta-analyses could dissolve some conflicts. The amount of the plants which have been asserted to possess anti-inflammatory effect is so much that evaluating all of them is out of the scope of this paper.

It should be noted that the word “natural anti-inflammatory” refers to natural compounds, lifestyle, exercise, and sleep and eating habits. There are numerous studies on natural compounds and herbal medicines issues but those outcomes are various and inconsistent; sometimes, the method of evoking extract has direct impact on the chemical constituents and it must be considered because the pharmacological effect of each medicinal herb is the result of plenty of metabolites combination and their synergistic effects; perhaps, it is one of the reasons of paradoxical results. In another aspect, considering side effects, contraindication, and pregnancy properties of plants is an important issue, which requires great caution on the part of the practitioner, but almost there is no reliable evidence about these. Further evidence-based studies and meta-analyses perhaps could create more clear vision and approach for the health professionals.

## Figures and Tables

**Table 1:** Mechanisms of anti-inflammatory action of the medicinal plants mentioned in this review article.

Herb	Inhibition of								
	TNF-*α*	COX-2	iNOS	NF-*κ*B	PGE_2_	NO	LOX	Complement	IFN-*γ*
*Curcuma longa*	*✓*	*✓*	*✓*				*✓*		
*Zingiber officinalis*	*✓*	*✓*				*✓*	*✓*		
*Rosmarinus officinalis*	*✓*							*✓*	
*Borago officinalis*	*✓*				*✓*				
*Oenothera biennis*	*✓*	*✓*				*✓*			*✓*
*Harpagophytum procumbens*	*✓*	*✓*	*✓*		*✓*				
*Boswellia serrata*	*✓*				*✓*	*✓*	*✓*	*✓*	*✓*
*Rosa canina*		*✓*			*✓*	*✓*	*✓*		
*Urtica dioica*		*✓*		*✓*					
*Uncaria tomentosa*	*✓*	*✓*	*✓*	*✓*	*✓*				
*Salvia officinalis*					*✓*		*✓*		
*Ribes nigrum*	*✓*	*✓*		*✓*	*✓*		*✓*		
*Persea americana*				*✓*					
*Glycine max*	*✓*	*✓*	*✓*		*✓*	*✓*			*✓*
*Elaeagnus angustifolia*	*✓*	*✓*							
*Vaccinium myrtillus*	*✓*		*✓*						
*Olea europaea*		*✓*	*✓*				*✓*		

Note: other mechanisms may also exist but we could not cover all of them.

## References

[1] Bagad A. S., Joseph J. A., Bhaskaran N., Agarwal A. (2013). Comparative evaluation of anti-inflammatory activity of curcuminoids, turmerones, and aqueous extract of *Curcuma longa*. *Advances in Pharmacological Sciences*.

[2] Ghasemian M., Owlia M. B. (2015). A different look at pulsed glucocorticoid protocols; is high dose oral prednisolone really necessary just after initiation of pulse therapy?. *Journal of Case Reports in Practice*.

[3] Nishiyama T., Mae T., Kishida H. (2005). Curcuminoids and sesquiterpenoids in turmeric (*Curcuma longa* L.) suppress an increase in blood glucose level in type 2 diabetic KK-Ay mice. *Journal of Agricultural and Food Chemistry*.

[4] Jurenka J. S. (2009). Anti-inflammatory properties of curcumin, a major constituent of *Curcuma longa*: a review of preclinical and clinical research. *Alternative Medicine Review*.

[5] Deodhar S. D., Sethi R., Srimal R. C. (1980). Preliminary study on antirheumatic activity of curcumin (diferuloyl methane). *The Indian Journal of Medical Research*.

[6] Lal B., Kapoor A. K., Asthana O. P. (1999). Efficacy of curcumin in the management of chronic anterior uveitis. *Phytotherapy Research*.

[7] Prucksunand C., Indrasukhsri B., Leethochawalit M., Hungspreugs K. (2001). Phase II clinical trial on effect of the long turmeric (*Curcuma longa* Linn) on healing of peptic ulcer. *The Southeast Asian Journal of Tropical Medicine and Public Health*.

[8] Bundy R., Walker A. F., Middleton R. W., Booth J. (2004). Turmeric extract may improve irritable bowel syndrome symptomology in otherwise healthy adults: a pilot study. *The Journal of Alternative and Complementary Medicine*.

[9] Shoskes D., Lapierre C., Cruz-Corerra M. (2005). Beneficial effects of the bioflavonoids curcumin and quercetin on early function in cadaveric renal transplantation: a randomized placebo controlled trial. *Transplantation*.

[10] Holt P. R., Katz S., Kirshoff R. (2005). Curcumin therapy in inflammatory bowel disease: a pilot study. *Digestive Diseases and Sciences*.

[11] Hanai H., Iida T., Takeuchi K. (2006). Curcumin maintenance therapy for ulcerative colitis: randomized, multicenter, double-blind, placebo-controlled trial. *Clinical Gastroenterology and Hepatology*.

[12] Heng M. C. Y., Song M. K., Harker J., Heng M. K. (2000). Drug-induced suppression of phosphorylase kinase activity correlates with resolution of psoriasis as assessed by clinical, histological and immunohistochemical parameters. *The British Journal of Dermatology*.

[13] Mahluji S., Ostadrahimi A., Mobasseri M., Attari V. E., Payahoo L. (2013). Anti-inflammatory effects of *Zingiber officinale* in type 2 diabetic patients. *Advanced Pharmaceutical Bulletin*.

[14] Ueda H., Ippoushi K., Takeuchi A. (2010). Repeated oral administration of a squeezed ginger (*Zingiber officinale*) extract augmented the serum corticosterone level and had anti-inflammatory properties. *Bioscience, Biotechnology and Biochemistry*.

[15] Drozdov V. N., Kim V. A., Tkachenko E. V., Varvanina G. G. (2012). Influence of a specific ginger combination on gastropathy conditions in patients with osteoarthritis of the knee or hip. *The Journal of Alternative and Complementary Medicine*.

[16] Khalvat A. (2005). Comparing the effects of ginger (*Zingiber officinale*) extract and ibuprofen on patients with osteoarthritis. *Archives of Iranian Medicine*.

[17] Haghighi A., Tavalaei N., Owlia M. B. (2006). Effects of ginger on primary knee osteoarthritis. *Indian Journal of Rheumatology*.

[18] Altman R. D., Marcussen K. C. (2001). Effects of a ginger extract on knee pain in patients with osteoarthritis. *Arthritis & Rheumatism*.

[19] Srivastava K. C., Mustafa T. (1992). Ginger (*Zingiber officinale*) in rheumatism and musculoskeletal disorders. *Medical Hypotheses*.

[20] Al-Sereiti M. R., Abu-Amer K. M., Sen P. (1999). Pharmacology of rosemary (*Rosmarinus officinalis* Linn.) and its therapeutic potentials. *Indian Journal of Experimental Biology*.

[21] Lukaczer D., Darland G., Tripp M. (2005). A pilot trial evaluating Meta050, a proprietary combination of reduced iso-alpha acids, rosemary extract and oleanolic acid in patients with arthritis and fibromyalgia. *Phytotherapy Research*.

[22] Sahu A., Rawal N., Pangburn M. K. (1999). Inhibition of complement by covalent attachment of rosmarinic acid to activated C3b. *Biochemical Pharmacology*.

[23] Amaral G. P., de Carvalho N. R., Barcelos R. P. (2013). Protective action of ethanolic extract of *Rosmarinus officinalis* L. in gastric ulcer prevention induced by ethanol in rats. *Food and Chemical Toxicology*.

[24] Nusier M. K., Bataineh H. N., Daradkah H. M. (2007). Adverse effects of rosemary (*Rosmarinus officinalis* L.) on reproductive function in adult male rats. *Experimental Biology and Medicine*.

[25] Abu-Al-Basal M. A. (2010). Healing potential of *Rosmarinus officinalis* L. on full-thickness excision cutaneous wounds in alloxan-induced-diabetic BALB/c mice. *Journal of Ethnopharmacology*.

[26] Dickmann L. J., Vandenbrink B. M., Lin Y. S. (2012). In vitro hepatotoxicity and cytochrome P450 induction and inhibition characteristics of carnosic acid, a dietary supplement with antiadipogenic properties. *Drug Metabolism and Disposition*.

[27] Miceli A., Aleo A., Corona O., Sardina M. T., Mammina C., Settanni L. (2014). Antibacterial activity of *Borago officinalis* and *Brassica juncea* aqueous extracts evaluated invitro and in situ using different food model systems. *Food Control*.

[28] Kast R. E. (2001). Borage oil reduction of rheumatoid arthritis activity may be mediated by increased cAMP that suppresses tumor necrosis factor-alpha. *International Immunopharmacology*.

[29] Soeken K. L., Miller S. A., Ernst E. (2003). Herbal medicines for the treatment of rheumatoid arthritis: a systematic review. *Rheumatology*.

[30] Foster R. H., Hardy G., Alany R. G. (2010). Borage oil in the treatment of atopic dermatitis. *Nutrition*.

[31] Montserrat-de la Paz S., García-Giménez M. D., Ángel-Martín M., Pérez-Camino M. C., Fernández Arche A. (2014). Long-chain fatty alcohols from evening primrose oil inhibit the inflammatory response in murine peritoneal macrophages. *Journal of Ethnopharmacology*.

[32] Montserrat-de La Paz S., Fernández-Arche M. A., Ángel-Martín M., García-Giménez M. D. (2014). Phytochemical characterization of potential nutraceutical ingredients from Evening Primrose oil (*Oenothera biennis* L.). *Phytochemistry Letters*.

[33] Montserrat-de la Paz S., Fernández-Arche Á., Ángel-Martín M., García-Giménez M. D. (2012). The sterols isolated from Evening Primrose oil modulate the release of proinflammatory mediators. *Phytomedicine*.

[34] Rezapour-Firouzi S., Arefhosseini S. R., Mehdi F. (2013). Immunomodulatory and therapeutic effects of Hot-nature diet and co-supplemented hemp seed, evening primrose oils intervention in multiple sclerosis patients. *Complementary Therapies in Medicine*.

[35] Belch J. J. F., Ansell D., Madhok R., O'dowd A., Sturrock R. D. (1988). Effects of altering dietary essential fatty acids on requirements for non-steroidal anti-inflammatory drugs in patients with rheumatoid arthritis: a double blind placebo controlled study. *Annals of the Rheumatic Diseases*.

[36] Brzeski M., Madhok R., Capell H. A. (1991). Evening primrose oil in patients with rheumatoid arthritis and side-effects of non-steroidal anti-inflammatory drugs. *British Journal of Rheumatology*.

[37] Jantti J., Seppala E., Vapaatalo H., Isomaki H. (1989). Evening primrose oil and olive oil in treatment of rheumatoid arthritis. *Clinical Rheumatology*.

[38] Setty A. R., Sigal L. H. (2005). Herbal medications commonly used in the practice of rheumatology: mechanisms of action, efficacy, and side effects. *Seminars in Arthritis and Rheumatism*.

[39] Huang T. H.-W., Tran V. H., Duke R. K. (2006). Harpagoside suppresses lipopolysaccharide-induced iNOS and COX-2 expression through inhibition of NF-*κ*B activation. *Journal of Ethnopharmacology*.

[40] Gyurkovska V., Alipieva K., Maciuk A. (2011). Anti-inflammatory activity of Devil’s claw *in vitro* systems and their active constituents. *Food Chemistry*.

[41] Fiebich B. L., Fiebich B. L., Heinrich M., Hiller K. O., Kammerer N. (2001). Inhibition of TNF-*α* synthesis in LPS-stimulated primary human monocytes by *Harpagophytum* extract SteiHap 69. *Phytomedicine*.

[42] Loew D., Möllerfeld J., Schrödter A., Puttkammer S., Kaszkin M. (2001). Investigations on the pharmacokinetic properties of *Harpagophytum* extracts and their effects on eicosanoid biosynthesis in vitro and ex vivo. *Clinical Pharmacology & Therapeutics*.

[43] Whitehouse L. W., Znamirowska M., Paul C. J. (1983). Devil's Claw (*Harpagophytum procumbens*): no evidence for anti-inflammatory activity in the treatment of arthritic disease. *Canadian Medical Association Journal*.

[44] McGregor G., Fiebich B., Wartenberg A., Brien S., Lewith G., Wegener T. (2005). Devil's claw (*Harpagophytum procumbens*): an anti-inflammatory herb with therapeutic potential. *Phytochemistry Reviews*.

[45] Grahame R., Robinson B. V. (1981). Devils's claw (*Harpagophytum procumbens*): pharmacological and clinical studies. *Annals of the Rheumatic Diseases*.

[46] Kimmatkar N., Thawani V., Hingorani L., Khiyani R. (2003). Efficacy and tolerability of Boswellia serrata extract in treatment of osteoarthritis of knee—a randomized double blind placebo controlled trial. *Phytomedicine*.

[47] Etzel R. (1996). Special extract of BOSWELLIA serrata (H15) in the treatment of rheumatoid arthritis. *Phytomedicine*.

[48] Madisch A., Miehlke S., Eichele O. (2008). Boswellia serrata extract for the treatment of collagenous colitis. A double-blind, randomized, placebo-controlled, multicenter trial. *International Journal of Phytotherapy & Phytopharmacology*.

[49] Houssen M. E., Ragab A., Mesbah A. (2010). Natural anti-inflammatory products and leukotriene inhibitors as complementary therapy for bronchial asthma. *Clinical Biochemistry*.

[50] Umar S., Umar K., Sarwar A. H. M. G. (2014). Boswellia serrata extract attenuates inflammatory mediators and oxidative stress in collagen induced arthritis. *Phytomedicine*.

[51] Gayathri B., Manjula N., Vinaykumar K. S., Lakshmi B. S., Balakrishnan A. (2007). Pure compound from *Boswellia serrata* extract exhibits anti-inflammatory property in human PBMCs and mouse macrophages through inhibition of TNF*α*, IL-1*β*, NO and MAP kinases. *International Immunopharmacology*.

[52] Kapil A., Moza N. (1992). Anticomplementary activity of Boswellic acids—an inhibitor of C3-convertase of the classical complement pathway. *International Journal of Immunopharmacology*.

[53] Ammon H. P. T. (2010). Modulation of the immune system by *Boswellia serrata* extracts and boswellic acids. *Phytomedicine*.

[54] Singh S., Khajuria A., Taneja S. C., Johri R. K., Singh J., Qazi G. N. (2008). Boswellic acids: a leukotriene inhibitor also effective through topical application in inflammatory disorders. *Phytomedicine*.

[55] Wenzig E. M., Widowitz U., Kunert O. (2008). Phytochemical composition and *in vitro* pharmacological activity of two rose hip (*Rosa canina* L.) preparations. *Phytomedicine*.

[56] Marstrand C., Warholm L., Kharazmi A., Winther K. (2013). The anti-inflammatory capacity of Rose-hip is strongly dependent on the seeds—a comparison of animal and human studies. *Osteoarthritis and Cartilage*.

[57] Rein E., Kharazmi A., Winther K. (2004). A herbal remedy, Hyben Vital (stand. powder of a subspecies of *Rosa canina* fruits), reduces pain and improves general wellbeing in patients with osteoarthritis—a double-blind, placebo-controlled, randomised trial. *Phytomedicine*.

[58] Winther K. (2014). Shells from rose-hip (*Rosa canina*) do not reduce symptom scores or improve anti-inflammatory property in patients with osteoarthritis—a double-blind, placebo-controlled, randomized study. *Osteoarthritis and Cartilage*.

[59] Willich S. N., Rossnagel K., Roll S. (2010). Rose hip herbal remedy in patients with rheumatoid arthritis—a randomised controlled trial. *Phytomedicine*.

[60] Kirkeskov B., Christensen R., Bügel S. (2011). The effects of rose hip (*Rosa canina*) on plasma antioxidative activity and C-reactive protein in patients with rheumatoid arthritis and normal controls: a prospective cohort study. *Phytomedicine*.

[61] Orhan D. D., Hartevioğlu A., Küpeli E., Yesilada E. (2007). In vivo anti-inflammatory and antinociceptive activity of the crude extract and fractions from *Rosa canina* L. fruits. *Journal of Ethnopharmacology*.

[62] Kharazmi A. (2008). Laboratory and preclinical studies on the anti-inflammatory and anti-oxidant properties of rosehip powder—identification and characterization of the active component GOPO^*Ⓡ*^. *Osteoarthritis and Cartilage*.

[63] Schwager J. P., Richard N., Wolfram S. (2008). 145 Anti-inflammatory and chondro-protecitve effects of rose hip powder and its constituent galactolipids gopo. *Osteoarthritis and Cartilage*.

[64] Johnson T. A., Sohn J., Inman W. D., Bjeldanes L. F., Rayburn K. (2013). Lipophilic stinging nettle extracts possess potent anti-inflammatory activity, are not cytotoxic and may be superior to traditional tinctures for treating inflammatory disorders. *Phytomedicine*.

[65] Chrubasik S., Enderlein W., Bauer R., Grabner W. (1997). Evidence for antirheumatic effectiveness of Herba Urticae dioicae in acute arthritis: a pilot study. *Phytomedicine*.

[66] Randall C., Randall H., Dobbs F., Hutton C., Sanders H. (2000). Randomized controlled trial of nettle sting for treatment of base-of-thumb pain. *Journal of the Royal Society of Medicine*.

[67] Shakibaei M., Allaway D., Nebrich S., Mobasheri A. (2012). Botanical extracts from rosehip (*Rosa canina*), willow bark (*Salix alba*), and nettle leaf (*Urtica dioica*) suppress IL-1*β*-induced NF-*κ*B activation in canine articular chondrocytes. *Evidence-Based Complementary and Alternative Medicine*.

[68] Riehemann K., Behnke B., Schulze-Osthoff K. (1999). Plant extracts from stinging nettle (*Urtica dioica*), an antirheumatic remedy, inhibit the proinflammatory transcription factor NF-*κ*B. *FEBS Letters*.

[69] Roschek B., Fink R. C., McMichael M., Alberte R. S. (2009). Nettle extract (*Urtica dioica*) affects key receptors and enzymes associated with allergic rhinitis. *Phytotherapy Research*.

[70] Aguilar J. L., Rojas P., Marcelo A. (2002). Anti-inflammatory activity of two different extracts of *Uncaria tomentosa* (Rubiaceae). *Journal of Ethnopharmacology*.

[71] Reis S. R. I. N., Valente L. M. M., Sampaio A. L. (2008). Immunomodulating and antiviral activities of *Uncaria tomentosa* on human monocytes infected with Dengue Virus-2. *International Immunopharmacology*.

[72] Piscoya J., Rodriguez Z., Bustamante S. A., Okuhama N. N., Miller M. J. S., Sandoval M. (2001). Efficacy and safety of freeze-dried cat's claw in osteoarthritis of the knee: mechanisms of action of the species *Uncaria guianensis*. *Inflammation Research*.

[73] Mur E., Hartig F., Eibl G., Schirmer M. (2002). Randomized double blind trial of an extract from the pentacyclic alkaloid-chemotype of *Uncaria tomentosa* for the treatment of rheumatoid arthritis. *The Journal of Rheumatology*.

[74] Sandoval-Chacón M., Thompson J. H., Zhang X.-J. (1998). Antiinflammatory actions of cat's claw: the role of NF-*κ*B. *Alimentary Pharmacology & Therapeutics*.

[75] Cisneros F. J., Jayo M., Niedziela L. (2005). An Uncaria tomentosa (cat's claw) extract protects mice against ozone-induced lung inflammation. *Journal of Ethnopharmacology*.

[76] Rojas-Duran R., González-Aspajo G., Ruiz-Martel C. (2012). Anti-inflammatory activity of mitraphylline isolated from *Uncaria tomentosa* bark. *Journal of Ethnopharmacology*.

[77] Rodrigues M. R. A., Kanazawa L. K. S., Neves T. L. M. D. (2012). Antinociceptive and anti-inflammatory potential of extract and isolated compounds from the leaves of *Salvia officinalis* in mice. *Journal of Ethnopharmacology*.

[78] Poeckel D., Greiner C., Verhoff M. (2008). Carnosic acid and carnosol potently inhibit human 5-lipoxygenase and suppress pro-inflammatory responses of stimulated human polymorphonuclear leukocytes. *Biochemical Pharmacology*.

[79] Bauer J., Kuehnl S., Rollinger J. M. (2012). Carnosol and carnosic acids from *Salvia officinalis* inhibit microsomal prostaglandin E2 synthase-1. *Journal of Pharmacology and Experimental Therapeutics*.

[80] Baricevic D., Sosa S., Della Loggia R. (2001). Topical anti-inflammatory activity of *Salvia officinalis* L. leaves: the relevance of ursolic acid. *Journal of Ethnopharmacology*.

[81] Carrasco F. R., Schmidt G., Romero A. L. (2009). Immunomodulatory activity of *Zingiber officinale* Roscoe, *Salvia officinalis* L. and *Syzygium aromaticum* L. essential oils: evidence for humor- and cell-mediated responses. *The Journal of Pharmacy and Pharmacology*.

[82] Halicioglu O., Astarcioglu G., Yaprak I., Aydinlioglu H. (2011). Toxicity of salvia officinalis in a newborn and a child: an alarming report. *Pediatric Neurology*.

[83] Wu D., Meydani M., Leka L. S. (1999). Effect of dietary supplementation with black currant seed oil on the immune response of healthy elderly subjects. *The American Journal of Clinical Nutrition*.

[84] Watson J., Byars M. L., McGill P., Kelman A. W. (1993). Cytokine and prostaglandin production by monocytes of volunteers and rheumatoid arthritis patients treated with dietary supplements of blackcurrant seed oil. *British Journal of Rheumatology*.

[85] Leventhal L. J., Boyce E. G., Zurier R. B. (1994). Treatment of rheumatoid arthritis with blackcurrant seed oil. *Rheumatology*.

[86] Ziboh V. A., Fletcher M. P. (1992). Dose-response effects of dietary *γ*-linolenic acid-enriched oils on human polymorphonuclear-neutrophil biosynthesis of leukotriene B4. *The American Journal of Clinical Nutrition*.

[87] Bishayee A., Thoppil R. J., Mandal A. (2013). Black currant phytoconstituents exert chemoprevention of diethylnitrosamine-initiated hepatocarcinogenesis by suppression of the inflammatory response. *Molecular Carcinogenesis*.

[88] Padilla-Camberos E., Martínez-Velázquez M., Flores-Fernández J. M., Villanueva-Rodríguez S. (2013). Acute toxicity and genotoxic activity of avocado seed extract (*Persea americana* Mill., c.v. *Hass*). *The Scientific World Journal*.

[89] Blotman F., Maheu E., Wulwik A., Caspard H., Lopez A. (1997). Efficacy and safety of avocado/soybean unsaponifiables in the treatment of symptomatic osteoarthritis of the knee and hip. A prospective, multicenter, three-month, randomized, double-blind, placebo-controlled trial. *Revue du Rhumatisme*.

[90] Ernst E. (2003). Avocado-soybean unsaponifiables (ASU) for osteoarthritis—a systematic review. *Clinical Rheumatology*.

[91] Maheu E., Cadet C., Marty M. (2014). Randomised, controlled trial of avocado-soybean unsaponifiable (Piascledine) effect on structure modification in hip osteoarthritis: the ERADIAS study. *Annals of the Rheumatic Diseases*.

[92] Jablonska S. (1998). Avocado/soybean unsaponifiables in the treatment of scleroderma: comment on the article by Maheu et al. *Arthritis & Rheumatism*.

[93] Borghi A., Corazza M., Minghetti S., Toni G., Virgili A. (2015). Avocado and soybean extracts as active principles in the treatment of mild-to-moderate vulvar lichen sclerosus: results of efficacy and tolerability. *Journal of the European Academy of Dermatology and Venereology*.

[94] Beigom Taheri J., Anbari F., Maleki Z., Boostani S., Zarghi A., Pouralibaba F. (2010). Efficacy of *Elaeagnus angustifolia* topical gel in the treatment of symptomatic oral lichen planus. *Journal of Dental Research, Dental Clinics, Dental Prospects*.

[95] Nikniaz Z., Ostadrahimi A., Mahdavi R., Ebrahimi A. A., Nikniaz L. (2014). Effects of *Elaeagnus angustifolia* L. supplementation on serum levels of inflammatory cytokines and matrix metalloproteinases in females with knee osteoarthritis. *Complementary Therapies in Medicine*.

[96] Ahmadiani A., Hosseiny J., Semnanian S. (2000). Antinociceptive and anti-inflammatory effects of *Elaeagnus angustifolia* fruit extract. *Journal of Ethnopharmacology*.

[97] Farahbakhsh S., Arbabian S., Emami F. (2011). Inhibition of cyclooxygenase type 1 and 2 enzyme by aqueous extract of *Elaeagnus angustifolia* in mice. *Basic and Clinical Neuroscience*.

[98] Nyman N. A., Kumpulainen J. T. (2001). Determination of anthocyanidins in berries and red wine by high-performance liquid chromatography. *Journal of Agricultural and Food Chemistry*.

[99] Kolehmainen M., Mykkänen O., Kirjavainen P. V. (2012). Bilberries reduce low-grade inflammation in individuals with features of metabolic syndrome. *Molecular Nutrition & Food Research*.

[100] Biedermann L., Mwinyi J., Scharl M. (2013). Bilberry ingestion improves disease activity in mild to moderate ulcerative colitis—an open pilot study. *Journal of Crohn's and Colitis*.

[101] Hoggard N., Cruickshank M., Moar K.-M. (2013). A single supplement of a standardised bilberry (*Vaccinium myrtillus* L.) extract (36% wet weight anthocyanins) modifies glycaemic response in individuals with type 2 diabetes controlled by diet and lifestyle. *Journal of Nutritional Science*.

[102] Bogani P., Galli C., Villa M., Visioli F. (2007). Postprandial anti-inflammatory and antioxidant effects of extra virgin olive oil. *Atherosclerosis*.

[103] Camargo A., Rangel-Zuñiga O. A., Haro C. (2014). Olive oil phenolic compounds decrease the postprandial inflammatory response by reducing postprandial plasma lipopolysaccharide levels. *Food Chemistry*.

[104] Najmi M., Vahdat Shariatpanahi Z., Tolouei M., Amiri Z. (2015). Effect of oral olive oil on healing of 10–20% total body surface area burn wounds in hospitalized patients. *Burns*.

[105] Sánchez-Fidalgo S., Villegas I., Cárdeno A. (2010). Extra-virgin olive oil-enriched diet modulates DSS-colitis-associated colon carcinogenesis in mice. *Clinical Nutrition*.

